# Changes in Maternal Platelet Physiology during Gestation and Their Interaction with Trophoblasts

**DOI:** 10.3390/ijms221910732

**Published:** 2021-10-03

**Authors:** Désirée Forstner, Jacqueline Guettler, Martin Gauster

**Affiliations:** Division of Cell Biology, Histology and Embryology, Gottfried Schatz Research Center, Medical University of Graz, 8010 Graz, Austria; jacqueline.serbin@medunigraz.at (J.G.); martin.gauster@medunigraz.at (M.G.)

**Keywords:** placenta, platelets, platelet-derived factors, trophoblasts, preeclampsia

## Abstract

Upon activation, maternal platelets provide a source of proinflammatory mediators in the intervillous space of the placenta. Therefore, platelet-derived factors may interfere with different trophoblast subtypes of the developing human placenta and might cause altered hormone secretion and placental dysfunction later on in pregnancy. Increased platelet activation, and the subsequent occurrence of placental fibrinoid deposition, are linked to placenta pathologies such as preeclampsia. The composition and release of platelet-derived factors change over gestation and provide a potential source of predicting biomarkers for the developing fetus and the mother. This review indicates possible mechanisms of platelet-trophoblast interactions and discusses the effect of increased platelet activation on placenta development.

## 1. Introduction

Anucleate platelets and their polyploid megakaryocyte progenitors in the bone marrow are only found in mammals. However, when looking at the evolution of mammalian platelets, it becomes apparent that neither live birth nor the presence of a placenta accounts for the evolution of platelets. Although the biological advantage gained from the presence of polyploid megakaryocytes and anucleate platelets has not been identified, a possible role for platelets during pregnancy in mammals has been suggested.

In humans, several large population-based studies suggest that the maternal platelet count decreases by approximately 10% in uncomplicated pregnancies at term, and rises again to basal levels postpartum [1]. The decrease in mean platelet count occurs gradually from first, to second, and third trimester [2], and is mediated by multiple physiological changes during pregnancy, including dilution of platelets by maternal blood plasma volume expansion and accelerated platelet sequestration and consumption in the placental circulation. In some cases, platelet counts drop below the lower limit [2,3], considered as incidental thrombocytopenia, which in some textbooks is referred to as gestational thrombocytopenia. Accordingly, two large cohort studies considered maternal platelet count below 116 × 10^9^/L (2.5th percentile) and 123 × 10^9^/L (5th percentile of normal platelet count in pregnant), respectively, as thrombocytopenic [3,4]. Pregnant women diagnosed with incidental thrombocytopenia are not at increased risk for a poor pregnancy outcome or delivery of a thrombocytopenic offspring [1,5]. Incidental thrombocytopenia usually resolves within a few days, up to a maximum of two months after delivery [6]. Therefore, otherwise healthy pregnant women diagnosed with mild thrombocytopenia after the mid second trimester are only carefully screened for the occurrence of hypertension and/or proteinuria.

For human pregnancy, both nonhemostatic as well as hemostatic platelet-dependent functions have been discussed to influence the placentation process. Nonhemostatic functions include promotion of trophoblast invasion by activating the chemokine receptor CCR1 in response to granule-stored CCR1 ligands such as CCL5 (also referred to as RANTES) and MIP-1α (macrophage inflammatory protein-1α or CCL3) [7]. Moreover, trophoblast invasion is enhanced by other platelet-derived factors such as epidermal growth factor (EGF), vascular endothelial growth factor (VEGF), and platelet-derived growth factor (PDGF) [8,9]. Recently, platelet-derived factors have been suggested to impair production and release of the crucial pregnancy hormone human chorionic gonadotropin (hCG) in early placenta [10].

On the other hand, the maternal coagulation system and platelets contribute to the generation of so-called perivillous fibrin-type fibrinoids, which can be detected at the surface of placental villi at sites where fibrinoid focally replaces the villous syncytiotrophoblast (SCT) [11]. Fibrin-type fibrinoid deposition can be considered as a normal process, starting very early in pregnancy, and fibrin deposits account for approximately 7% of the villous surface at term [12]. Interestingly, continuous deposition and breakdown of perivillous fibrin-type fibrinoid have been discussed as an important regulator of intervillous hemodynamics, shaping the villous trees and the intervillous space (IVS) [11]. Accordingly, unfavourable villous branching would lead to maternal blood coagulation in areas where turbulences, or even local stasis, occur, which in turn would result in subsequent degeneration of these newly formed sprouts. Both nonhemostatic as well as hemostatic platelet-dependent functions may affect placenta development and its physiology very early in gestation. This assumption is based on the fact that maternal platelets were found on the surface of the placental villi and in intercellular gaps of trophoblast cell columns from gestational week 5 onwards [10,13]. Whether or not these platelet-dependent effects are good or bad for the placenta depends on the extent of their activation [14]. Here, we summarize current views on how maternal platelets can interact with the placental trophoblast subtypes and the consequences the release of platelet-derived factors at the maternal-fetal interface may have.

## 2. Platelet-Derived Factors

### 2.1. Types of Granules

Due to the platelets’ ability to become activated in response to biochemical or mechanical stimuli, such as the exposure of the subendothelial extracellular matrix at site of vascular injury, they play an essential role in wound healing [15]. Upon platelet activation, their highly organized cytoskeleton converts the disc-shaped platelets into hemisphere-shaped structures with extended filopodia [16]. Platelet activation consequently results in firm adhesion, aggregation and the formation of a hemostatic platelet plug [17]. Beside their role in hemostasis, they are also key players in inflammatory processes [15]. Anucleate platelets are equipped with a variety of adhesion molecules, coagulation factors, chemokines and cytokines, which are released upon activation [16,18].

**Figure 1:**
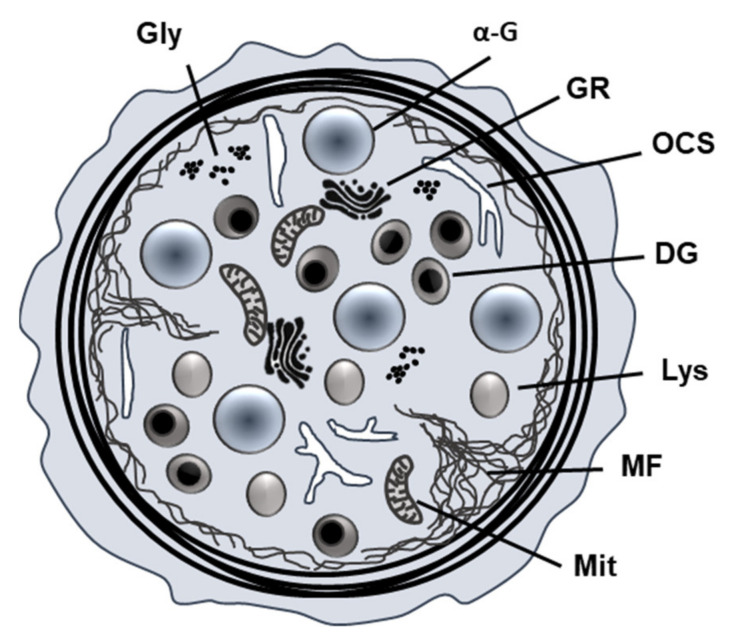
*Schematic presentation of platelet granules.* Platelets possess alpha-granules, dense granules and lysosomes. Abbreviations: Gly: glycogen, α-G: α-granules, DG: dense granules, Lys: lysosomes, GR: Golgi remnants, MF: microfilaments, Mit: Mitochondria, OCS: open canalicular system; Figure according to Neumüller et al., 2014 [20].

Platelet-derived factors are stored in three types of intercellular secretory organelles, including lysosomes, alpha-granules and dense granules (also referred to as dense bodies) (Figure 1). Alpha-granules and dense granules are only found in platelets and megakaryocytes, whereas lysosomes are ubiquitous [1]. These three secretory granules mainly differ in molecular content, size and their abundance [16]. Alpha-granules, with a density of about 80 granules per platelet, are the most abundant ones and have a size of about 200–500 nm [1].

Beside membrane-bound proteins that become exposed to the platelet surface, such as integrin αIIbβ3 and αVβ3, or the leucine-rich repeat family receptors (e.g., GPIb-IX-V complex), alpha-granules also store soluble proteins, such as platelet factor 4 (PF4), von Willebrand Factor (vWF) or vascular endothelial growth factor (VEGF) that are released into the extracellular space [19]. In general, the granule content of alpha-granules can be divided according to their functions into coagulation factors, chemokines, adhesion molecules, immunological molecules and regulators of growth and angiogenesis, which are summarized in Table 1 [18]. Alpha-granules release their content upon exposure of platelets to strong agonists such as thrombin, but also to weak agonists such as adenosine diphosphate (ADP) [16].

Human platelets contain about three to eight dense granules per platelet, with a slight acidic lumen [21]. Dense granules are known to store many low molecular weight compounds, such as calcium, adenosine triphosphate (ATP), ADP or serotonin, which are potent activators of platelet aggregation and vasoconstriction and are released during exocytosis [16,18]. Serotonin accumulates in dense granules by active uptake from the plasma [21]. The lysosomes are morphologically similar to the alpha-granules [16] and contain hydrolases, cathepsins and lysosomal membrane proteins [18], and are mainly activated by strong agonists such as thrombin [16].

### 2.2. Platelet Releasate

The platelet releasate (PR) is defined as a cocktail of soluble and vesicular (exosomes and microparticles) signals, which is released from platelets upon activation into the external milieu [22]. A wide variety of adhesive and soluble agonists induces platelet activation through their respective receptors [23]. Recent data suggest that the platelet releasate may be adapted to its environment and thus is altered in a state of inflammation and disease [22,24,25]. It has been reported, that in cardiovascular diseases, platelet secretion is increased [1].

Parsons et al. identified 894 different proteins released from thrombin-induced platelets from 32 healthy adult humans, of which 277 proteins were reproducibly found in every donor and, therefore, defined as core releasate proteins [22]. Dependent on the strength of the platelet agonist, the platelet response ranges from shape change and platelet activation up to release of platelet granule content [16]. The PR basically comprises molecules such as growth factors, coagulation proteins, cytokines, proinflammatory molecules and adhesion molecules, which can either act in an autocrine or in a paracrine manner [16].

### 2.3. Platelet-Derived Extracellular Vesicles

Since the platelet releasate is not only defined as a mixture of soluble factors, but also of vesicular signals [22], platelet-derived extracellular vesicles (P-EVs) have to be taken into account as biologically active mediators [26]. Upon activation, platelets are capable of releasing extracellular products into the external milieu [27] due to membrane blebbing and subsequent shedding of microvesicles [28]. P-EVs are the most abundant EVs in the human blood [26] and were first described as “platelet dust” by Wolf in 1967 [29]. Double-layer phospholipid membrane vesicles have gained importance in a broad range of research fields in the last couple of years and have been defined as a heterogeneous pool of vesicles referred to as exosomes, microvesicles or extracellular vesicles (EVs) [28,30,31].

In order to further distinguish them in size, studies have described and defined different centrifugation protocols that define the microvesicles to be isolated at 10,000–20,000× *g* and the exosomes to be isolated by centrifugation at 100,000–200,000× *g* [28]. The cargo of platelet-derived extracellular vesicles is considered to consist of cytosolic and membrane proteins as well as messenger RNA (mRNA), circular RNA (circRNA), noncoding RNA (lncRNA) and microRNA (miRNA) [26]. Although the distinguishing criteria for EVs in the blood are still unclear, markers such as CD31, CD41, CD42 and P-Selectin are widely used as markers for P-EVs [26]. Regarding their procoagulant function, platelet-derived microvesicles are more likely considered as procoagulatory active mediators than the smallest of all platelet-derived vesicles, the exosomes. They have a size of 40–120 nm [32] and might lack factor X, prothrombin and annexin-V [26,33].

## 3. Interaction of Maternal Platelets and Trophoblasts

### 3.1. Implantation and Development of the Placenta

A successful implantation of the embryo into the uterine endometrium begins at day 6–7 post fertilization, with the polar blastocyst attaching to the endometrium. At this time point, the blastocyst is composed of two cell lineages, the outlying trophectoderm (TE) and the inner cell mass, with the latter mass facing towards the maternal side upon attachment [14]. Thereafter, the blastocyst breaks through the uterine luminal epithelium and invades the maternal endometrial stromal cells (ESC) [14]. Prior to this process, ESCs differentiate from elongated, fibroblast-like mesenchymal cells to rounded, epithelioid-like cells, a process defined as decidualization [34]. In nonpregnant women, this cyclic regulated process is divided into a proliferative and a secretory phase. Upon implantation of the blastocyst, the ESCs supply the embryo with nutrients, prevent rejection of the blastocyst by the immune system and take part in the regulation of trophoblast invasion. The extracellular matrix of the decidua interacts with the trophoblasts during invasion, which results in the production of fibronectin, laminin and collagen type IV [35].

The initial syncytium is formed due to the fusion processes of the TE, which then starts to invade into the maternal decidua, resulting in complete embedment of the blastocyst at day 14 post fertilization. This primary syncytium then starts to develop lacunae, which are fluid-filled spaces that later develop into the IVS. The primary syncytium is organized into trabeculae. Throughout the subsequent villous stage of placental development, cytotrophoblasts rapidly proliferate to invade the trabeculae of the primary syncytium. The thereby formed primary villi consist of an inner core of cytotrophoblasts with a surrounding SCT. The following stage of placental development includes the generation of the secondary villi. These secondary villi develop by invasion of fetal mesenchymal cells into the prior formed primary villi. Sequentially, tertiary villi are formed by emergence of fetal vessels within the villi core [14,36].

Thereafter, cytotrophoblasts penetrate the primary syncytium and generate contact with the maternal decidua. A multilayered structure develops through reorganization of cytotrophoblasts, tailing in the formation of trophoblast cell columns [37]. These maintain their proliferative stem cell character, whereas the cells that loose contact with the basement membrane and invade towards the decidua as an invasive cell type, are characterized as extravillous trophoblasts (EVT) [36,37].

Different EVT subtypes have been described. The endovascular EVT (eEVT) directs along the spiral artery, whereas the interstitial EVT (iEVT) moves towards luminal structures such as spiral arteries, uterine veins, and lymphatics, by migration through the decidual interstitium. The iEVT is involved in the remodeling of spiral arteries, and also interacts with decidual stroma cells. This is of great importance for the attachment of the placenta to the uterus [14,36]. During this early time of gestation, the developing embryo is nourished histotrophically by glandular secretion products [38]. The endoglandular EVTs invade uterine glands, and have the ability to replace the glandular epithelium and connect the glands to the IVS. Before the IVS is filled with maternal blood, the SCT is in direct contact with maternal blood plasma. Therefore, the nutrition of the early human placenta and fetus is by a combination of maternal blood plasma and glandular secretion products [36,38].

### 3.2. Remodeling of Uterine Spiral Arteries

The IVS is perfused by the maternal spiral arteries. These are tightly coiled vessels, that arise from the uterine arteries of the mother. Due to invasion of EVTs the maternal spiral arteries are remodeled early in gestation, and the EVTs dilate the vessels at the entrance to the IVS into low resistance wide pore vessels [39]. The remodeled arteries lose their vasoactivity due to a loss of actin in the smooth muscle cells that surround the arteries. This results in a constant blood flow into the IVS, even when the maternal blood pressure increases [36].

Besides remodeling the spiral arteries, EVTs also invade the lumen and plug the spiral arteries during the first trimester of pregnancy. Therefore, maternal blood cells are obstructed to flow into the IVS [40]. Maternal blood cells are trapped within the plugs, allowing only an ultrafiltrate of the maternal blood to pass into the IVS [37]. The prevention of oxygenated blood flow into the IVS is hypothesized to be of great importance for successful placental development and trophoblast differentiation during early gestation by creating a beneficial and physiologically hypoxic environment [41]. A study by Roberts et al. (2017) suggests perfusion through the spiral arteries of the IVS from gestational age (GA) 6–7 onwards [39]. Sharp-bordered channels within the spiral artery trophoblastic plugs are described from GA 7 onwards. This leads to the assumption that maternal blood flow into the IVS is not completely obstructed by EVT plugs [39]. From the end of first trimester the flow is described as constant, as during this time the extravillous trophoblast plugs dissolve and the constant and velocity reduced blood flow into the IVS is established by the remodeled spiral arteries in a healthy pregnancy [39,42]. However, recent findings suggest that trophoblast plug disintegration is not completed by the end of the first trimester, but immunohistochemical staining indicated a partial persistence until mid-gestation. The rapid increase in blood flow into the IVS that was observed by end of first trimester can, however, be traced back to a dimensional increase of the arteries [43].

### 3.3. Route of Platelets into the Intervillous Space/EVTs in First and Term

As mentioned above, EVTs invade the lumen of the spiral arteries from the beginning of gestation onwards, obstructing maternal blood flow into the IVS by plug formation [40].

**Figure 2:**
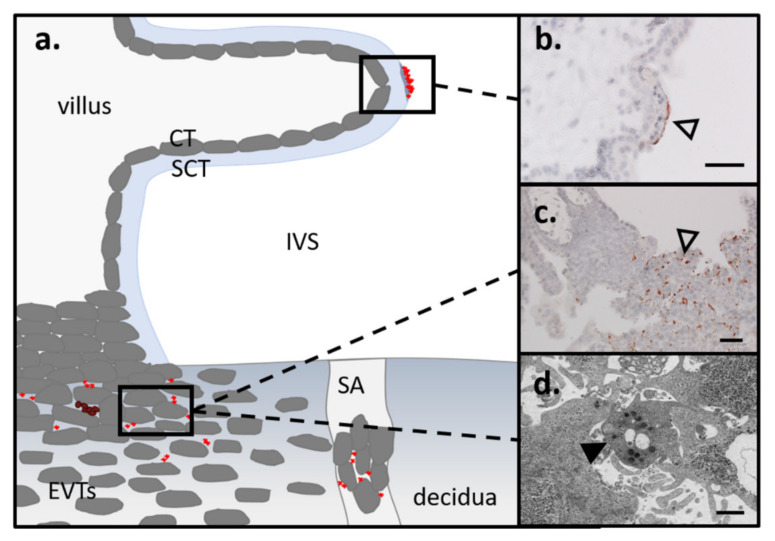
Interaction of maternal platelets with trophoblasts in the first trimester placenta. (**a**) Scheme of first trimester anchoring villus shows the location of maternal platelets based on recent immunohistochemistry and transmission electron microscopy (TEM) data. With immunohistochemistry for platelet marker CD42b, platelets were found (open arrowheads) on (**b**) perivillous fibrin depositions and (**c**) in intercellular gaps between EVTs of anchoring trophoblast columns. (**d**) The presence of platelets between EVTs was further verified by TEM (platelet shown by arrowhead). Scale bars of b and c represent 50 µm. Scale bar of d represents 1 µm. EVT: extravillous trophoblast; IVS: intervillous space; CT: cytotrophoblast; SCT: syncytiotrophoblast; SA: spiral artery.

However, with a diameter of 2–3 µm, platelets are the smallest cells of human blood. Recent immunohistochemical studies of first trimester villous placental tissue led to the assumption that maternal platelets are the first blood cells entering into the IVS (Figure 2a). The platelets were found adhering to the surface of the villous SCT or initial villous fibrinoid deposit (Figure 2b), and were also found between EVTs in anchoring parts of trophoblast cell columns (Figure 2c) [10,14,44]. The adherence of maternal platelets in very early stages of gestation seems to be a normal process, since in an immunohistochemical survey of over 30 first trimester placental tissues, 93.6% of all cases showed platelets on the surface of placental villi. In the same cohort, the appearance of maternal platelets between EVTs in distal trophoblast cell column interstices was described in almost 80% of all cases [10]. Whether the degree of platelets, either on the surface of villous trophoblasts or in interstices of EVTs, is already altered in the first trimester of pregnancies manifesting in pregnancy pathology later on, remains to be answered. 

An electronic microscopic survey showed filopodia formation, as well as fine-grained material in the OCS, in intercellular gaps of distal EVT column parts, indicating considerable platelet activation (Figure 2d). These findings suggest that maternal plasma components, including particles such as platelets, can leak into paracellular trophoblast gaps and enter the IVS through this alternative route to the spiral arteries from early gestation onwards [13].

## 4. Mechanisms of Platelet Activation

### 4.1. Agonist-Induced Activation

**Figure 3:**
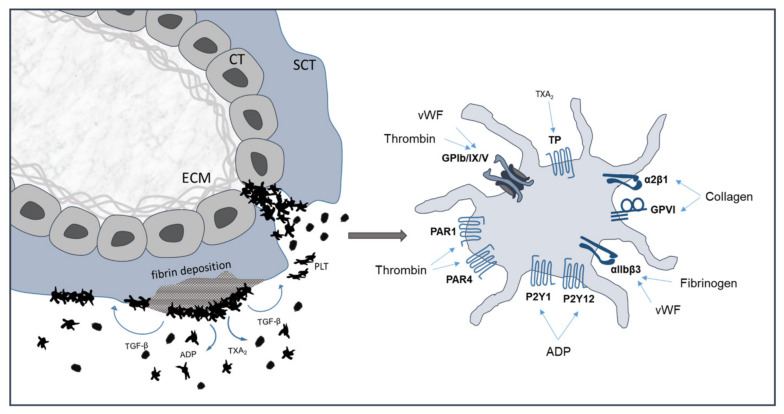
Platelet activation at the maternal-fetal interface. Scheme of a first trimester placental villus shows adherent platelets on fibrin deposition and on extracellular matrix on areas of disrupted villous trophoblasts. Activated platelet show relevant surface receptors. CT: cytotrophoblast; SCT: syncytiotrophoblast; ECM: extracellular matrix; PLT: platelet; TXA_2_: thromboxane A_2_; ADP: adenosine diphosphate; TGF-β: transforming growth factor beta; vWF: von Willebrand factor; TP: thromboxane receptor.

There are multiple pathways contributing to platelet activation, granule release and platelet adherence [45,46,47,48]. ADP is stored in the dense granules at high concentrations and is released from adherent platelets upon activation. ADP contributes to platelet activation by binding to purinergic receptors P_2_Y_1_ and P_2_Y_12_ (Figure 3). The agonist is involved in protective hemostasis as well as occlusive thrombus formation [49]. Another agonist, Thromboxane A_2_ (TXA_2_), is released from adherent platelets and enhances the recruitment and aggregation of additional platelets to the primary plug by binding to TPα and TPβ receptors. It activates platelets during both protective hemostasis and pathologic thrombus formation [49]. Platelets express several collagen receptors in the form of membrane glycoproteins (GP) and integrins. They play a key role in hemostasis and, upon vessel damage, they can interact with the extracellular matrix. Of these GP receptors, GPIb-V-IX is important for tethering of the platelet to collagen via vWF, whereas GPVI is required for collagen-induced platelet activation. GPVI is a member of the immunoglobulin superfamily type I transmembrane glycoproteins. It takes part in procoagulatory activity and is involved in the subsequent formation of thrombin and fibrin [23,50]. Thrombin is the most powerful platelet activator and can activate platelets at very low concentrations [51,52]. It binds and activates the protease-activated receptor (PAR)-1 on the platelet surface [53,54,55,56,57]. PAR-4 is also expressed by human platelets, but requires higher concentrations of thrombin for activation [54]. Thrombin also binds GPIb, which has been proposed to enhance the specificity of thrombin activation of PAR-1 [58]. Other contributing factors are, for example, serotonin, which helps to recruit the platelets to the site of injury, and epinephrine, which plays a supplementary role that is overlapping with the P_2_Y_12_ receptor signaling [49].

### 4.2. Platelet Activation Due to Mechanical Stimuli (Shear Stress)

As platelets are subjected to an unsteady and probably continuously changing shear stress in the IVS, the assumption of platelet activation upon shear stress and turbulence is very important in platelet-trophoblast interaction. Platelet activation regarding shear stress has been the subject of many nonplacenta-related research projects over the past decades.

A recent study by Roka-Moiia et al. showed that platelets exposed to continuous shear stress, but not to biochemical agonists, exhibited an increase of phosphatidylserine externalization (PSE) and procoagulant activity. In the study, markers of platelet activation (P-selectin and integrin αIIbβ3 activation) and apoptosis (mitochondrial membrane potential, caspase 3 activation and PSE) were examined. Biochemical agonists such as ADP and thrombin are potent inducers of αIIbβ3 activation and/or P-selectin exposure. No integrin αIIbβ3 activation occurred upon shear stress exposure, and P-selectin levels remained nearly unchanged. Shear-mediated platelet activation induced a different pattern of platelet surface activation markers, with enhanced PSE and thrombin generation on the platelet surface [59].

Platelet activation under steady shear stress has been studied intensively over the past decades in regard to shear stress-induced unfolding of vWF and its binding to platelet receptor GPIb, which induced a significant activation of the platelets upon a specific threshold [60,61,62,63].

Recent findings by Pushin et al. also described an analytical approach to platelet activation under unsteady shear stress. The approach was based on the idea that under unsteady flow, the conformation of vWF molecules on the platelet surface are dynamically changing and unfolding. The efficient interaction of vWF and multiple GPIb receptors on the platelet surface should increase and the platelet was assumed to be primed for activation [64].

### 4.3. Platelets in Pregnancy

During normotensive healthy pregnancies, a decrease in platelet count occurs, with between 4.4% and 11.6% developing gestational thrombocytopenia (defined as a platelet count below 150 × 10^9^/L) [6]. These platelet distributions are most likely related to hemodilution, i.e., a higher plasma volume during gestation and a possible increased platelet clearance. Furthermore, the mean platelet volume and the platelet volume distribution width is increased in pregnant women compared to nonpregnant women [65]. Platelets from pregnant women are also hyper-responsive to activation during gestation [66,67]. Increasing basal P-selectin levels are found in platelet-derived microparticles during gestation, indicating platelet activation [68]. Furthermore, plasma levels of β-thromboglobulin (β TG) and PF4, secreted from platelet α-granules, and adenosine secreted from platelet dense granules, are also elevated during pregnancy, suggesting increased platelet activation and release of granule content [69,70,71]. In addition, the concentration of TXA_2_ observed in normotensive pregnancies is increased above levels reported in normal healthy nonpregnant women [72]. However, the mechanisms underlying platelet activation in pregnancy remain largely unknown. Nevertheless, these studies suggest an underlying physiological balance during pregnancy to prime platelets for activation, while other reports show regulation of thrombosis due to the suppression by Pregnancy-Specific Glycoproteins (PSGs) [73], which are members of the immunoglobulin superfamily. In human gestation, PSGs are expressed and released by the SCT. They induce the release of anti-inflammatory cytokines (e.g., IL-10 and TGFβ1) from monocytes, macrophages, and other cells. Human PSG1 binds αIIbβ3 and inhibits platelet-fibrinogen interaction. Human PSG9 also has inhibitory properties. In species with hemochorial placentation, in which maternal blood cells are in direct contact with trophoblasts, high expression of PSGs reflect a requirement of immunoregulation in the maternal circulation. This may be necessary to inhibit platelet aggregation and thrombosis in the prothrombotic maternal environment of human gestation [73].

Studies suggest not only different levels of platelet activation during pregnancy but also an altered protein content of the platelet releasate (PR). Szklanna et al. investigated the profile of PRs of 18 women with healthy pregnancies and 13 nonpregnant women. Of 723 identified proteins in the PR, 69 of these proteins were found to be altered in platelet releasate from pregnant women. This includes proteins that are only expressed during pregnancy, such as PSGs and human placental lactogen. Moreover, the population of exosomal vesicles present in the PR is also modified in pregnancy such that the mode size and the particle/mL size are decreased. This demonstrates that platelets and their released cargo are different in physiological stressful situations such as pregnancy. This may represent a promising beginning to understand possible roles of platelet activation in pregnancy complications [74].

## 5. Pro and Anticoagulatory Mechanisms of the Placenta

### 5.1. Coagulatory Mechanism of the Trophoblasts

Pregnancy is accompanied with remarkable changes in hemostasis towards hypercoagulability [75] and hypofibrinolysis, due to decreased fibrinolytic activity [76]. Major key players in the regulation of hemostasis are thrombomodulin (TM) and tissue factor, which are both known to be expressed by the SCT [77,78]. Tissue factor, which is exposed on the cell membrane at the site of injury, is a central regulator of the so-called extrinsic pathway of blood coagulation, as it initiates the cascade by binding circulating factor VII [79]. This in turn leads to the activation of factor X, which triggers the generation of thrombin and subsequently converts fibrinogen to fibrin [79].

A study from Sood et al. revealed a differentiation-dependent gene expression program in murine trophoblasts that confers a thromboresistant phenotype onto these cells [80]. In order to keep the hemostatic balance in the placenta, the transmembrane glycoprotein thrombomodulin is a ligand for thrombin and prevents increased coagulation in the placenta [81]. The binding of thrombomodulin to thrombin activates protein C, which in turn builds a complex with protein S and thus degrades factor Va and factor VIIIa to finally reduce thrombin formation [77].

### 5.2. Subepithelial Extracellular Matrix Exposed upon Damaged Syncytiotrophoblast 

Beside the expression of anti- and procoagulatory mediators, the fibrinolytic system is very important in sustaining a healthy balance in the coagulation system of the placenta. Pregnancy is described as a status of hypofibrinolysis, with remarkable changes in hemostasis, such as an increase of clotting factors and coagulability and a decrease of anticoagulants and fibrinolytic activity [76]. The development of intrauterine growth restriction (IUGR) and preeclampsia (PE) is often accompanied with disturbances in the fibrinolytic system [76]. 

The major key player in the fibrinolytic system are the plasminogen activators urokinase and tissue type plasminogen activator (uPA/tPA), with their corresponding inhibitors the plasminogen activator inhibitor type 1 (PAI-1) and type 2 (PAI-2) [82]. The degradation of noncellular components, the extracellular matrix (ECM), is mainly inhibited by the action of PAI-1, which is upregulated in wound healing and in fibrotic tissue [82].

Overexpression of PAI-1 has been described to increase fibrin accumulation and insufficient placentation [76]. Towards term, placental fibrin depositions increase and make up about 7% of the villous surface at term [11]. Placenta insufficiency due to vessel occlusion and infarcts, because of increased fibrin accumulation, might even lead to late fetal loss [77].

Placental pathologies, such as PE, are associated with an upregulation of PAI-1 in the placenta, as well as with elevated plasma levels. These results suggest that localized elevated levels of PAI-1 may play a role in thrombotic complications. Limited information is available on the factors that regulate the production of PAI-1 within healthy and pathological placentas, but cytokines or growth factors, such as TGF-β, could be key players [77].

Disruption of the SCT, for example as a result of enhanced blood flow velocity and jet-like streams surrounded by turbulence, is replaced by the fibrin-type fibrinoid, which is defined as a product of the coagulation cascade and resulting in the so-called perivillous fibrinoid [11,12,83]. The fibrin-type fibrinoid mainly consists of fibrin, whereas the matrix-type fibrinoid is secreted by the EVT trophoblast and is mainly composed of glycoproteins and collagen type IV [11].

The plasminogen inhibitors are both expressed in the cytoplasm of the CT and the SCT, whereas the SCT also expresses PAI-1 and PAI-2 in its plasma membrane [76]. Trophoblast invasion is accompanied by degradation of the extracellular matrix, whereas the expression of PAI-1 in the invasive EVT may prevent excessive invasion into maternal tissue [76]. Interestingly, it has been reported that PAI-1 promotes tumor cell immigration, while it inhibits trophoblast invasion [84].

Once exposed to maternal blood, the trophoblastic basal lamina, which contains collagen, fibronectin and laminin, has procoagulant potential, and this suggests that focal degeneration of the SCT results in local blood clotting [12,85].

Collagen, as a major component of the extracellular matrix, provides structural support [86], but also triggers platelet activation and the formation of a hemostatic plug [87].

Platelets possess several collagen receptors, of which α2β1integrin and the immunoglobulin superfamily member GPVI are the most prominent [87]. The binding of vWF to the platelet surface glycoprotein Ib-V-IX complex is important for the initial binding to exposed collagen. The activated platelets subsequently release further procoagulant factors, such as ADP or TXA_2_, to tighten the binding of integrin α2β1to collagen, and increases the affinity of integrin αIIβ3 to fibrinogen [88].

### 5.3. Coagulatory Factors Released by Trophoblasts into the Maternal Circulation

Trophoblasts release a diverse spectrum of hormones, growth factors and extracellular vesicles (EV) from the first trimester onwards [89,90]. The amount of released EVs increases with ongoing gestation, and pathological pregnancies show a further significant increase [91,92]. EVs are shed from the SCT into the IVS and, therefore, directly into the maternal blood circulation. Thus, maternal cells, such as endothelial cells, circulating immune cells and platelets, are exposed and affected by the diverse cargo of fetal material [93,94].

Different vesicle fractions transport a diverse repertoire of placenta-derived molecules, which could affect maternal cells in various ways [95]. Hence, the complex maternal-fetal cross-talk is greatly shaped by syncytiotrophoblast-derived EVs (STBEV) [93]. During healthy pregnancy, placenta-derived EVs are continuously released into the maternal blood circulation and thus prime the maternal immune system to the ongoing pregnancy. However, this also presents a challenging task for the maternal immune system [93,96,97].

For instance, the release of syncytiotrophoblast-derived EVs is elevated in PE compared to healthy pregnancies [92,96,98]. The size and cargo of proteins present within these EVs are also significantly altered [99]. Platelets isolated from women who develop PE in later stages of gestation show an increased reactivity, display elevated platelet activation marker CD63 on the cells surface, and reveal an increase in platelet-bound fibrinogen [66].

STBEVs interact with platelets and affect their function. Furthermore, they increase the rate and size of thrombus formation in vitro in whole blood under fluidic flow. In general, STBEVs from patients with PE have an increased effect on platelet activation and function, implying that they could contribute to the increased thrombotic risk of PE. The difference in STBEVs composition in PE compared to healthy pregnancies may correlate with the heterogeneity of maternal symptoms [100].

## 6. Platelet Activation in Pregnancy Complications

### 6.1. Preeclampsia

Alterations in the coagulation and fibrinolytic system have been linked to pregnancy pathologies such as PE [101]. With a prevalence of about 2–8% of all pregnancies, PE is one of the most common pregnancy complications in which the placenta plays an essential role. Although the major cause for the development of PE is still unknown, generally the placenta as a potential major cause is being taken into account [102]. Mothers, diagnosed with PE suffer from hypertension (≥140/≥90 mmHg) and proteinuria (≥300 mg/24 h), which are accompanied with a higher risk for preterm birth, including all associated risks for the mother and the new-born [103]. Depending on the GA of delivery, PE is categorized into early-onset PE (before the 34 weeks of gestation) and late-onset PE (after 34 weeks of gestation) [102].

However, PE provides a broad spectrum of phenotypes with heterogeneous etiology. A study from Than et al. uncovered altered abundance of proteins of the coagulation cascade in maternal proteomics in patients who subsequently developed preterm or term PE. Of note, in preterm cases of PE the extent of changes was larger than in term PE cases [104]. A very important mechanism in the development of PE might be a disturbed turnover of the SCT. An unbalanced trophoblast fusion and increased turnover rates, which result in increased release of apoptotic material into the maternal circulation, might provoke a systemic inflammatory response in the mother and might contribute to the development of PE [105]. STBEVs isolated from preeclamptic pregnancies have a higher tissue factor activity, and thus a higher capacity to stimulate platelet activation, than those STBEVs isolated from healthy pregnancies [100,106].

In general, changes in platelet function, coagulation and thrombotic factors are strongly associated with the onset of PE [107]. PE has been described with increased platelet aggregation, but also with a significant increased mean platelet volume (MPV) compared to healthy pregnant control cases [101]. The molecular basis of the onset of PE is still not clear, but some studies have linked increased thromboxane A_2_ (TXA_2_) production, and hence the consequences of platelet activation, to the onset of PE. Low-dose aspirin (<150 mg daily), as an inhibitor of the cyclooxygenase, is widely used as prophylaxis for PE [108]. A study from Tannetta et al. showed that aspirin blocked PE derived STBEV-induced platelet aggregation, providing a potential explanation for the beneficial effect of low-dose aspirin treatment [100]. PE is often accompanied with increased endothelial damage, which may lead to increased fibrin deposition, platelet consumption and thrombotic microangiopathy (TMA) [109], which is defined as of a group of diseases characterized by microangiopathatic hemolysis, thrombocytopenia and microthrombi formation [110]. TMA is developed in 10–20% of severe PE cases, leading to endothelial dysfunction and altered production of circulating factors, such as vWF, thrombomodulin, fibronectin and PAI-1. These mechanisms further induce hypercoagulability and platelet consumption. In turn, activated platelets release more thromboxane A2, contributing to the vicious cycle that further causes endothelial injury [111].

### 6.2. Treatments

For pregnancies with a high-risk of developing PE, the administration of low-dose aspirin from very early stages of gestation onwards is commonly advised. [112], leading to the assumption that anti-platelet therapy has a protective effect on pathological blood coagulation in the placenta [113]. However, the action of aspirin to prevent PE is poorly understood, and mechanisms such as “aspirin resistance” have to be considered [112].

A study from Roberge et al. described a significant reduction in the prevalence of PE, and a dose-response effect for the prevention of PE, when low dose aspirin (50–150 mg/day) was administered before the 16th week of gestation [114]. However, a study from Stern et al. showed an inhibitory effect of aspirin on platelet aggregation in a proportion of pregnant women, but also demonstrated that patients respond in an individual and dose-dependent manner [112].

Alternatively, low-molecular weight heparin or prasugrel is used as an anticoagulant substance during pregnancy. Low-molecular-weight heparin (LMWH) does not cross the placenta and has also been described as a promising therapy for especially severe placenta-mediated pregnancy complications [115]. Prasugrel is a third generation thienopyridine that blocks P_2_Y_12_ similar to clopidogrel, which is the most commonly used thienopyridine, and has been recently introduced into clinical practice. A case report from Tello-Montoliu described a successful pregnancy outcome with prasugrel therapy. However, the use of P_2_Y_12_ receptor inhibitors during pregnancy is still under debate [116]. 

## 7. Outlook/Conclusions

Platelets have been found to carry a complex and dynamic transcriptome, similar to that of many nucleated cells. They are equipped with a complex transcriptome of mRNA, miRNA, long noncoding RNA, pre-mRNA and circular RNA. The majority of transcripts in platelets are already synthesized by megakaryocytes during thrombopoiesis, and only a small number are acquired through cell-cell transfer while circulating in the blood. Since platelets have an active spliceosome, and can process mRNA, proteins are synthesized and might play a role in platelet response, function and in cell-cell interactions (e.g., by P-EVs) [117,118].

Studies have shown dynamic changes in the transcriptome of platelets in response to pathogens, stressors or inflammatory signals. A set of the most abundant transcripts is even comparable between species, such as human and murine platelets. A study from Middleton et al. revealed that platelets from septic patients, and platelets from appropriate septic mouse models, undergo similar changes in the transcripts of platelets [119]. Hence, it is tempting to speculate on a change of the transcriptome of platelets during healthy and diseased pregnancy. Furthermore, it has been shown that inflammation induces variable transcripts in individuals [117]. Specific sets of genetic signatures might serve as promising biomarkers for predicting pathologies, such as PE, and their clinical outcomes [118]. It is well established that human and murine platelets show significant morphological differences. Murine platelets are of smaller size, bigger in number and have a greater granule heterogeneity [120]. A study by Balkenhol et al. also found transcriptome-based variations between human and murine platelets. These variations refer to the expression of different mRNA and protein in platelet activation, as well as modulation [121]. Nonetheless, because of many other similarities in platelet biology and the generally well-conserved central cascade, mice are considered an excellent in vivo model for various study designs of platelet physiology [120,121]. Nevertheless, with work on the murine model, some technical issues must be taken into consideration. A general standardization for experiments such as the tail bleeding time assay, or the examination of vessels in vivo after injury (with e.g., ferric chloride), is of great importance for comparison of results. In addition, time and costs of breeding an appropriate in vivo model must be contemplated [122].

In conclusion, platelets are underestimated contributors to healthy development during pregnancy. Platelets at the fetal-maternal interface are a source of proinflammatory mediators, which might interact with different trophoblast subtypes of the developing placenta. Platelets possess the capability to undergo dynamic changes leading to an altered platelet releasate during pregnancy and a diverse set of transcripts in disease. These findings underline the importance of platelet subtypes (and their cargo) as promising biomarkers in the future.

## Figures and Tables

**Table 1:** List of platelet-derived factors. Factors are stored in dense bodies, lysosomes and α-granules. Factors stored in α-granules are distinguished by their function into regulators of growth and angiogenesis, coagulation factors, chemokines, adhesion molecules and immunologic molecules, according to Burnouf et al. [18].

α-Granules		Lysosomes	Dense Granules
**Adhesion Molecules**	**Coagulation Factors**	α-Arabinoside	ATP
P-Selectin	Factor V	β-Galactosidase	ADP
von Willebrand factor	Factor XI	β-Glucuronidase	Serotonin
Vitronectin	Factor XIII	n-Acetylglucosaminidase	Ca++
Fibrinogen	Prothrombin	Elastase	Epinephrine
Integrin αIIbβ3	Antithrombin	Collagenase	Histamine
Integrin αVβ3	α2-Macroglobulin	Cathepsin	
Fibronectin	α2-Antiplasmin		
	Plasmin, Plasminogen		
	Protein S		
	PAI-1, TFPI		
**Regulators of Growth and Angiogenesis**	**Immunological Molecules**		
bFGF	Complement factors		
HGF	Platelet factor H		
IGF-1	β1H Globulin		
VEGF-A, -C	Factor D		
PDGF-AA, -AB, -BB	C1 Inhibitor		
BDNF	IgG		
Angiostatin	Thymosin-β4		
PF4			
Thrombospondin			
EGF			
CTGF			
TGF-β	**Chemokines**		
Angiopoietin-1	IL-8		
SDF-1	NAP-2		
MMP-1, -2, -9	RANTES		
Endostatin	MCP-1,-3		
TIMP-1, -4	MIP-1α		
BMP-2, -4, -6	β-Thromboglobulin		

## Abbreviations

ADP	Adenosine diphosphate
ATP	Adenosine triphosphate
α-G	α-granules
β TG	β-thromboglobulin
BDNF	Brain Derived Neurotrophic Factor
bFGF	Basic Fibroblast Growth Factor;
BMP	Bone Morphogenetic Protein;
C	Complement
CTGF	Connective Tissue Growth Factor
circRNA	Circular RNA
CT	Cytotrophoblast
DG	Dense granules
ECM	Extracellular matrix
EGF	Epidermal growth factor
EV	Extracellular vesicle
EVT	Extravillous trophoblast
eEVT	Endovascular trophoblast
ESC	Endometrial stromal cells
GA	Gestational age
Gly	Glycogen
GP	Glycoprotein
GPIIb/IIIa	Glycoproteins IIb/IIIa
HGF	Hepatocyte growth factor
hCG	Human chorionic gonadotropin
iEVT	Interstitial extravillous trophoblast
IGF-1	Insulin-like Growth Factor-1
IL-8	Interleukin 8
IUGR	Intrauterine growth restriction
IVS	Intervillous space
Lys	Lysosomes
lncRNA	Noncoding RNA
MCP-1	Monocyte Chemotactic Protein-1
MCP-3	Monocyte Chemotactic Protein-3
MIP-1α	Macrophage Inflammatory Protein 1α
MMP	Matrix Metalloproteinase
MF	Microfilaments
miRNA	MicroRNA
Mit	Mitochondria
MPV	Mean platelet volume
mRNA	Messenger RNA
NAP-2	Neutrophil-Activating Protein-2
OCS	Open canicular system
PAI-1	Plasminogen Activator Inhibitor
PDGF	Platelet-derived growth factor
PE	Preeclampsia
PF4	Platelet factor 4
PLT	Platelet
P-MVs	Platelet-derived microvesicles
PR	Platelet releasate
PSE	Phosphatidylserine externalization
PSG	Pregnancy-Specific Glycoprotein
RANTES	Regulated on Activation, Normal T Cell Expressed and Secreted
SDF-1	Stromal Cell-Derived Factor-1
TFPI	Tissue Factor Pathway Inhibitor
SA	Spiral artery
SS	Shear stress
SCT	Syncytiotrophoblast
STBEV	Syncytiotrophoblast-derived extracellular vesicles
TE	Trophectoderm
TGF-β	Transforming Growth Factor-β
TIMP	Tissue Inhibitor of Metalloproteinases
TM	Thrombomodulin
TMA	Thrombotic microangiopathy
tPA	Tissue-type plasminogen activator
TXA2	Thromboxane A2
uPA	Urokinase-type plasminogen activator
VEGF	Vascular endothelial growth factor
vWF	Von Willebrand factor

## References

[1] Michelson A.D. (2013). Platelets.

[2] Reese J.A., Peck J.D., Yu Z., Scordino T.A., Deschamps D.R., McIntosh J.J., Terrell D.R., Vesely S.K., George J.N. (2019). Platelet sequestration and consumption in the placental intervillous space contribute to lower platelet counts during pregnancy. Am. J. Hematol..

[3] Boehlen F., Hohlfeld P., Extermann P., Perneger T.V., de Moerloose P. (2000). Platelet count at term pregnancy: A reappraisal of the threshold. Obstet. Gynecol..

[4] Sainio S., Kekomäki R., Riikonen S., Teramo K. (2000). Maternal thrombocytopenia at term: A population-based study. Acta Obstet. Gynecol. Scand..

[5] Shehata N., Burrows R., Kelton J.G. (1999). Gestational thrombocytopenia. Clin. Obstet. Gynecol..

[6] Cines D.B., Levine L.D. (2017). Thrombocytopenia in pregnancy. Hematol. Am. Soc. Hematol. Educ. Program.

[7] Sato Y., Fujiwara H., Zeng B.-X., Higuchi T., Yoshioka S., Fujii S. (2005). Platelet-derived soluble factors induce human extravillous trophoblast migration and differentiation: Platelets are a possible regulator of trophoblast infiltration into maternal spiral arteries. Blood.

[8] Bass K.E., Morrish D., Roth I., Bhardwaj D., Taylor R., Zhou Y., Fisher S.J. (1994). Human cytotrophoblast invasion is up-regulated by epidermal growth factor: Evidence that paracrine factors modify this process. Dev. Biol..

[9] Lash G.E., Warren A.Y., Underwood S., Baker P.N. (2003). Vascular endothelial growth factor is a chemoattractant for trophoblast cells. Placenta.

[10] Forstner D., Maninger S., Nonn O., Guettler J., Moser G., Leitinger G., Pritz E., Strunk D., Schallmoser K., Marsche G. (2020). Platelet-derived factors impair placental chorionic gonadotropin beta-subunit synthesis. J. Mol. Med. Berl. Ger..

[11] Kaufmann P., Huppertz B., Frank H.-G. (1996). The fibrinoids of the human placenta: Origin, composition and functional relevance. Ann. Anat. Anat. Anz..

[12] Nelson D.M., Crouch E.C., Curran E.M., Farmer D.R. (1990). Trophoblast interaction with fibrin matrix. Epithelialization of perivillous fibrin deposits as a mechanism for villous repair in the human placenta. Am. J. Pathol..

[13] Guettler J., Forstner D., Cvirn G., Maninger S., Brugger B.A., Nonn O., Kupper N., Pritz E., Wernitznig S., Dohr G. (2021). Maternal platelets pass interstices of trophoblast columns and are not activated by HLA-G in early human pregnancy. J. Reprod. Immunol..

[14] Moser G., Guettler J., Forstner D., Gauster M. (2019). Maternal Platelets—Friend or Foe of the Human Placenta?. Int. J. Mol. Sci..

[15] Holinstat M. (2017). Normal platelet function. Cancer Metastasis Rev..

[16] Coppinger J.A., Maguire P.B. (2007). Insights into the platelet releasate. Curr. Pharm. Des..

[17] De Meyer S.F. (2017). Platelet granules in vascular integrity. Blood.

[18] Burnouf T., Strunk D., Koh M.B.C., Schallmoser K. (2016). Human platelet lysate: Replacing fetal bovine serum as a gold standard for human cell propagation?. Biomaterials.

[19] Blair P., Flaumenhaft R. (2009). Platelet alpha-granules: Basic biology and clinical correlates. Blood Rev..

[20] Neumüller J., Ellinger A., Wagner T. (2015). Transmission Electron Microscopy of Platelets FROM Apheresis and Buffy-Coat-Derived Platelet Concentrates.

[21] McNicol A., Israels S.J. (1999). Platelet dense granules: Structure, function and implications for haemostasis. Thromb. Res..

[22] Parsons M.E.M., Szklanna P.B., Guerrero J.A., Wynne K., Dervin F., O’Connell K., Allen S., Egan K., Bennett C., McGuigan C. (2018). Platelet Releasate Proteome Profiling Reveals a Core Set of Proteins with Low Variance between Healthy Adults. Proteomics.

[23] Li Z., Delaney M.K., O’Brien K.A., Du X. (2010). Signaling during platelet adhesion and activation. Arterioscler. Thromb. Vasc. Biol..

[24] Guerrero J.A., Bennett C., van der Weyden L., McKinney H., Chin M., Nurden P., McIntyre Z., Cambridge E.L., Estabel J., Wardle-Jones H. (2014). Gray platelet syndrome: Proinflammatory megakaryocytes and α-granule loss cause myelofibrosis and confer metastasis resistance in mice. Blood.

[25] Deppermann C., Cherpokova D., Nurden P., Schulz J.-N., Thielmann I., Kraft P., Vögtle T., Kleinschnitz C., Dütting S., Krohne G. (2013). Gray platelet syndrome and defective thrombo-inflammation in Nbeal2-deficient mice. J. Clin. Investig..

[26] Tao S.-C., Guo S.-C., Zhang C.-Q. (2017). Platelet-derived Extracellular Vesicles: An Emerging Therapeutic Approach. Int. J. Biol. Sci..

[27] Taus F., Meneguzzi A., Castelli M., Minuz P. (2019). Platelet-Derived Extracellular Vesicles as Target of Antiplatelet Agents. What Is the Evidence?. Front. Pharmacol..

[28] Szatanek R., Baran J., Siedlar M., Baj-Krzyworzeka M. (2015). Isolation of extracellular vesicles: Determining the correct approach (Review). Int. J. Mol. Med..

[29] Wolf P. (1967). The nature and significance of platelet products in human plasma. Br. J. Haematol..

[30] Aatonen M., Grönholm M., Siljander P.R.-M. (2012). Platelet-derived microvesicles: Multitalented participants in intercellular communication. Semin. Thromb. Hemost..

[31] Margolis L., Sadovsky Y. (2019). The biology of extracellular vesicles: The known unknowns. PLoS Biol..

[32] Nederveen J.P., Warnier G., Di Carlo A., Nilsson M.I., Tarnopolsky M.A. (2020). Extracellular Vesicles and Exosomes: Insights From Exercise Science. Front. Physiol..

[33] Heijnen H.F., Schiel A.E., Fijnheer R., Geuze H.J., Sixma J.J. (1999). Activated platelets release two types of membrane vesicles: Microvesicles by surface shedding and exosomes derived from exocytosis of multivesicular bodies and alpha-granules. Blood.

[34] Burton G.J., Jauniaux E. (2015). What is the placenta?. Am. J. Obstet. Gynecol..

[35] Okada H., Tsuzuki T., Murata H. (2018). Decidualization of the human endometrium. Reprod. Med. Biol..

[36] Turco M.Y., Moffett A. (2019). Development of the human placenta. Dev. Camb. Engl..

[37] Huppertz B., Gauster M., Orendi K., König J., Moser G. (2009). Oxygen as modulator of trophoblast invasion. J. Anat..

[38] Moser G., Gauster M., Orendi K., Glasner A., Theuerkauf R., Huppertz B. (2010). Endoglandular trophoblast, an alternative route of trophoblast invasion? Analysis with novel confrontation co-culture models. Hum. Reprod. Oxf. Engl..

[39] Roberts V.H.J., Morgan T.K., Bednarek P., Morita M., Burton G.J., Lo J.O., Frias A.E. (2017). Early first trimester uteroplacental flow and the progressive disintegration of spiral artery plugs: New insights from contrast-enhanced ultrasound and tissue histopathology. Hum. Reprod. Oxf. Engl..

[40] Weiss G., Sundl M., Glasner A., Huppertz B., Moser G. (2016). The trophoblast plug during early pregnancy: A deeper insight. Histochem. Cell Biol..

[41] James J.L., Stone P.R., Chamley L.W. (2006). The regulation of trophoblast differentiation by oxygen in the first trimester of pregnancy. Hum. Reprod. Update.

[42] Brugger B.A., Guettler J., Gauster M. (2020). Go with the Flow-Trophoblasts in Flow Culture. Int. J. Mol. Sci..

[43] Allerkamp H.H., Clark A.R., Lee T.C., Morgan T.K., Burton G.J., James J.L. (2021). Something old, something new: Digital quantification of uterine vascular remodelling and trophoblast plugging in historical collections provides new insight into adaptation of the utero-placental circulation. Hum. Reprod. Oxf. Engl..

[44] Blaschitz A., Siwetz M., Schlenke P., Gauster M. (2015). Adhering maternal platelets can contribute to the cytokine and chemokine cocktail released by human first trimester villous placenta. Placenta.

[45] Brass L.F. (2003). Thrombin and platelet activation. Chest.

[46] Davì G., Patrono C. (2007). Platelet activation and atherothrombosis. N. Engl. J. Med..

[47] Varga-Szabo D., Pleines I., Nieswandt B. (2008). Cell adhesion mechanisms in platelets. Arterioscler. Thromb. Vasc. Biol..

[48] Offermanns S. (2006). Activation of platelet function through G protein-coupled receptors. Circ. Res..

[49] Jennings L.K. (2009). Mechanisms of platelet activation: Need for new strategies to protect against platelet-mediated atherothrombosis. Thromb. Haemost..

[50] Nieswandt B., Pleines I., Bender M. (2011). Platelet adhesion and activation mechanisms in arterial thrombosis and ischaemic stroke. J. Thromb. Haemost..

[51] Mann K.G. (2003). Thrombin formation. Chest.

[52] Brummel K.E., Paradis S.G., Butenas S., Mann K.G. (2002). Thrombin functions during tissue factor-induced blood coagulation. Blood.

[53] Vu T.K., Hung D.T., Wheaton V.I., Coughlin S.R. (1991). Molecular cloning of a functional thrombin receptor reveals a novel proteolytic mechanism of receptor activation. Cell.

[54] Coughlin S.R. (2005). Protease-activated receptors in hemostasis, thrombosis and vascular biology. J. Thromb. Haemost..

[55] Leger A.J., Covic L., Kuliopulos A. (2006). Protease-activated receptors in cardiovascular diseases. Circulation.

[56] Landis R.C. (2007). Protease activated receptors: Clinical relevance to hemostasis and inflammation. Hematol. Oncol. Clin. N. Am..

[57] Martorell L., Martínez-González J., Rodríguez C., Gentile M., Calvayrac O., Badimon L. (2008). Thrombin and protease-activated receptors (PARs) in atherothrombosis. Thromb. Haemost..

[58] De Candia E., Hall S.W., Rutella S., Landolfi R., Andrews R.K., De Cristofaro R. (2001). Binding of thrombin to glycoprotein Ib accelerates the hydrolysis of Par-1 on intact platelets. J. Biol. Chem..

[59] Roka-Moiia Y., Walk R., Palomares D.E., Ammann K.R., Dimasi A., Italiano J.E., Sheriff J., Bluestein D., Slepian M.J. (2020). Platelet Activation via Shear Stress Exposure Induces a Differing Pattern of Biomarkers of Activation versus Biochemical Agonists. Thromb. Haemost..

[60] Shankaran H., Alexandridis P., Neelamegham S. (2003). Aspects of hydrodynamic shear regulating shear-induced platelet activation and self-association of von Willebrand factor in suspension. Blood.

[61] Moake J.L., Turner N.A., Stathopoulos N.A., Nolasco L.H., Hellums J.D. (1986). Involvement of large plasma von Willebrand factor (vWF) multimers and unusually large vWF forms derived from endothelial cells in shear stress-induced platelet aggregation. J. Clin. Invest..

[62] Goto S., Salomon D.R., Ikeda Y., Ruggeri Z.M. (1995). Characterization of the unique mechanism mediating the shear-dependent binding of soluble von Willebrand factor to platelets. J. Biol. Chem..

[63] Zhang C., Kelkar A., Neelamegham S. (2019). von Willebrand factor self-association is regulated by the shear-dependent unfolding of the A2 domain. Blood Adv..

[64] Pushin D.M., Salikhova T.Y., Zlobina K.E., Guria G.T. (2020). Platelet activation via dynamic conformational changes of von Willebrand factor under shear. PLoS ONE.

[65] Freitas L.G., Alpoim P.N., Komatsuzaki F., das Carvalho M.G., Dusse L.M.S. (2013). Preeclampsia: Are platelet count and indices useful for its prognostic?. Hematol. Amst. Neth..

[66] Janes S.L., Goodall A.H. (1994). Flow cytometric detection of circulating activated platelets and platelet hyper-responsiveness in pre-eclampsia and pregnancy. Clin. Sci. Lond. Engl..

[67] Sheu J.-R., Hsiao G., Lin W.-Y., Chen T.-F., Chien Y.-Y., Lin C.-H., Tzeng C.-R. (2002). Mechanisms involved in agonist-induced hyperaggregability of platelets from normal pregnancy. J. Biomed. Sci..

[68] Lok C.A.R., Nieuwland R., Sturk A., Hau C.M., Boer K., Vanbavel E., Vanderpost J.A.M. (2007). Microparticle-associated P-selectin reflects platelet activation in preeclampsia. Platelets.

[69] Ayhan A., Akkök E., Urman B., Yarali H., Dündar S., Kirazli S. (1990). Beta-thromboglobulin and platelet factor 4 levels in pregnancy and preeclampsia. Gynecol. Obstet. Invest..

[70] Hayashi M., Kiumi F., Mitsuya K. (1999). Changes in platelet ATP secretion and aggregation during pregnancy and in preeclampsia. Am. J. Med. Sci..

[71] Yoneyama Y., Suzuki S., Sawa R., Otsubo Y., Power G.G., Araki T. (2000). Plasma adenosine levels increase in women with normal pregnancies. Am. J. Obstet. Gynecol..

[72] Fitzgerald D.J., Mayo G., Catella F., Entman S.S., FitzGerald G.A. (1987). Increased thromboxane biosynthesis in normal pregnancy is mainly derived from platelets. Am. J. Obstet. Gynecol..

[73] Shanley D.K., Kiely P.A., Golla K., Allen S., Martin K., O’Riordan R.T., Ball M., Aplin J.D., Singer B.B., Caplice N. (2013). Pregnancy-specific glycoproteins bind integrin αIIbβ3 and inhibit the platelet-fibrinogen interaction. PLoS ONE.

[74] Szklanna P.B., Parsons M.E., Wynne K., O’Connor H., Egan K., Allen S., Ní Áinle F., Maguire P.B. (2019). The Platelet Releasate is Altered in Human Pregnancy. Proteomics Clin. Appl..

[75] Hellgren M. (2003). Hemostasis during normal pregnancy and puerperium. Semin. Thromb. Hemost..

[76] Ye Y., Vattai A., Zhang X., Zhu J., Thaler C.J., Mahner S., Jeschke U., von Schönfeldt V. (2017). Role of Plasminogen Activator Inhibitor Type 1 in Pathologies of Female Reproductive Diseases. Int. J. Mol. Sci..

[77] Lanir N., Aharon A., Brenner B. (2003). Procoagulant and anticoagulant mechanisms in human placenta. Semin. Thromb. Hemost..

[78] Kohli S., Singh K.K., Gupta A., Markmeyer P., Lochmann F., Gupta D., Rana R., Elwakiel A., Huebner H., Ruebner M. (2021). Placental thromboinflammation impairs embryonic survival by reducing placental thrombomodulin expression. Blood.

[79] Mackman N. (2009). The role of tissue factor and factor VIIa in hemostasis. Anesth. Analg..

[80] Sood R., Kalloway S., Mast A.E., Hillard C.J., Weiler H. (2006). Fetomaternal cross talk in the placental vascular bed: Control of coagulation by trophoblast cells. Blood.

[81] Loghmani H., Conway E.M. (2018). Exploring traditional and nontraditional roles for thrombomodulin. Blood.

[82] Urano T., Suzuki Y., Iwaki T., Sano H., Honkura N., Castellino F.J. (2019). Recognition of Plasminogen Activator Inhibitor Type 1 as the Primary Regulator of Fibrinolysis. Curr. Drug Targets.

[83] Burton G.J., Woods A.W., Jauniaux E., Kingdom J.C.P. (2009). Rheological and physiological consequences of conversion of the maternal spiral arteries for uteroplacental blood flow during human pregnancy. Placenta.

[84] Fitzpatrick T.E., Graham C.H. (1998). Stimulation of plasminogen activator inhibitor-1 expression in immortalized human trophoblast cells cultured under low levels of oxygen. Exp. Cell Res..

[85] Benirschke K., Burton G.J., Baergen R.N. (2000). Pathology of the Human Placenta.

[86] Oefner C.M., Sharkey A., Gardner L., Critchley H., Oyen M., Moffett A. (2015). Collagen type IV at the fetal-maternal interface. Placenta.

[87] Nieswandt B., Watson S.P. (2003). Platelet-collagen interaction: Is GPVI the central receptor?. Blood.

[88] Farndale R.W. (2006). Collagen-induced platelet activation. Blood Cells. Mol. Dis..

[89] Mincheva-Nilsson L., Baranov V. (2014). Placenta-derived exosomes and syncytiotrophoblast microparticles and their role in human reproduction: Immune modulation for pregnancy success. Am. J. Reprod. Immunol. N. Y..

[90] Kshirsagar S.K., Alam S.M., Jasti S., Hodes H., Nauser T., Gilliam M., Billstrand C., Hunt J.S., Petroff M.G. (2012). Immunomodulatory molecules are released from the first trimester and term placenta via exosomes. Placenta.

[91] Goswami D., Tannetta D.S., Magee L.A., Fuchisawa A., Redman C.W.G., Sargent I.L., von Dadelszen P. (2006). Excess syncytiotrophoblast microparticle shedding is a feature of early-onset pre-eclampsia, but not normotensive intrauterine growth restriction. Placenta.

[92] Knight M., Redman C.W., Linton E.A., Sargent I.L. (1998). Shedding of syncytiotrophoblast microvilli into the maternal circulation in pre-eclamptic pregnancies. Br. J. Obstet. Gynaecol..

[93] Tong M., Chamley L.W. (2015). Placental extracellular vesicles and feto-maternal communication. Cold Spring Harb. Perspect. Med..

[94] Kupper N., Huppertz B. (2021). The endogenous exposome of the pregnant mother: Placental extracellular vesicles and their effect on the maternal system. Mol. Aspects Med..

[95] Tong M., Kleffmann T., Pradhan S., Johansson C.L., DeSousa J., Stone P.R., James J.L., Chen Q., Chamley L.W. (2016). Proteomic characterization of macro-, micro- and nano-extracellular vesicles derived from the same first trimester placenta: Relevance for feto-maternal communication. Hum. Reprod. Oxf. Engl..

[96] Germain S.J., Sacks G.P., Sooranna S.R., Soorana S.R., Sargent I.L., Redman C.W. (2007). Systemic inflammatory priming in normal pregnancy and preeclampsia: The role of circulating syncytiotrophoblast microparticles. J. Immunol..

[97] Giacomini E., Alleva E., Fornelli G., Quartucci A., Privitera L., Vanni V.S., Viganò P. (2019). Embryonic extracellular vesicles as informers to the immune cells at the maternal-fetal interface. Clin. Exp. Immunol..

[98] Redman C.W.G., Tannetta D.S., Dragovic R.A., Gardiner C., Southcombe J.H., Collett G.P., Sargent I.L. (2012). Review: Does size matter? Placental debris and the pathophysiology of pre-eclampsia. Placenta.

[99] Pillay P., Moodley K., Moodley J., Mackraj I. (2017). Placenta-derived exosomes: Potential biomarkers of preeclampsia. Int. J. Nanomed..

[100] Tannetta D.S., Hunt K., Jones C.I., Davidson N., Coxon C.H., Ferguson D., Redman C.W., Gibbins J.M., Sargent I.L., Tucker K.L. (2015). Syncytiotrophoblast Extracellular Vesicles from Pre-Eclampsia Placentas Differentially Affect Platelet Function. PLoS ONE.

[101] Thalor N., Singh K., Pujani M., Chauhan V., Agarwal C., Ahuja R. (2019). A correlation between platelet indices and preeclampsia. Hematol. Transfus. Cell Ther..

[102] Huppertz B. (2018). The Critical Role of Abnormal Trophoblast Development in the Etiology of Preeclampsia. Curr. Pharm. Biotechnol..

[103] Huppertz B., Schleußner E. (2018). Die Plazenta: Grundlagen und Klinische Bedeutung.

[104] Than N.G., Romero R., Tarca A.L., Kekesi K.A., Xu Y., Xu Z., Juhasz K., Bhatti G., Leavitt R.J., Gelencser Z. (2018). Integrated Systems Biology Approach Identifies Novel Maternal and Placental Pathways of Preeclampsia. Front. Immunol..

[105] Gauster M., Moser G., Orendi K., Huppertz B. (2009). Factors involved in regulating trophoblast fusion: Potential role in the development of preeclampsia. Placenta.

[106] Tannetta D., Masliukaite I., Vatish M., Redman C., Sargent I. (2017). Update of syncytiotrophoblast derived extracellular vesicles in normal pregnancy and preeclampsia. J. Reprod. Immunol..

[107] Ness R.B., Sibai B.M. (2006). Shared and disparate components of the pathophysiologies of fetal growth restriction and preeclampsia. Am. J. Obstet. Gynecol..

[108] Tallah A., Lecarpentier E., Goffinet F., Doret-Dion M., Gaucherand P., Tsatsaris V. (2017). Aspirin for Prevention of Preeclampsia. Drugs.

[109] Preedy V.R. (2011). Handbook of Growth and Growth Monitoring in Health and Disease.

[110] Lokki A.I., Heikkinen-Eloranta J. (2021). Pregnancy induced TMA in severe preeclampsia results from complement-mediated thromboinflammation. Hum. Immunol..

[111] Baumwell S., Karumanchi S.A. (2007). Pre-eclampsia: Clinical manifestations and molecular mechanisms. Nephron Clin. Pract..

[112] Stern C., Mayer-Pickel K., Weiss E.-C., Kutllovci-Hasani K., Nanda M., Eberhard K., Cervar-Zivkovic M., Prüller F. (2021). Low Dose Aspirin in high-risk pregnancies: The volatile effect of acetylsalicylic acid on the inhibition of platelets uncovered by G. Born’s light transmission aggregometry. J. Reprod. Immunol..

[113] Roberge S., Nicolaides K., Demers S., Hyett J., Chaillet N., Bujold E. (2017). The role of aspirin dose on the prevention of preeclampsia and fetal growth restriction: Systematic review and meta-analysis. Am. J. Obstet. Gynecol..

[114] Roberge S., Bujold E., Nicolaides K.H. (2018). Aspirin for the prevention of preterm and term preeclampsia: Systematic review and metaanalysis. Am. J. Obstet. Gynecol..

[115] Rodger M.A., Gris J.-C., de Vries J.I.P., Martinelli I., Rey É., Schleussner E., Middeldorp S., Kaaja R., Langlois N.J., Ramsay T. (2016). Low-molecular-weight heparin and recurrent placenta-mediated pregnancy complications: A meta-analysis of individual patient data from randomised controlled trials. Lancet Lond. Engl..

[116] Tello-Montoliu A., Seecheran N.A., Angiolillo D.J. (2013). Successful pregnancy and delivery on prasugrel treatment: Considerations for the use of dual antiplatelet therapy during pregnancy in clinical practice. J. Thromb. Thrombolysis.

[117] Davizon-Castillo P., Rowley J.W., Rondina M.T. (2020). Megakaryocyte and Platelet Transcriptomics for Discoveries in Human Health and Disease. Arterioscler. Thromb. Vasc. Biol..

[118] Fisher M.H., Di Paola J. (2018). Genomics and transcriptomics of megakaryocytes and platelets: Implications for health and disease. Res. Pract. Thromb. Haemost..

[119] Middleton E.A., Rowley J.W., Campbell R.A., Grissom C.K., Brown S.M., Beesley S.J., Schwertz H., Kosaka Y., Manne B.K., Krauel K. (2019). Sepsis alters the transcriptional and translational landscape of human and murine platelets. Blood.

[120] Schmitt A., Guichard J., Massé J.M., Debili N., Cramer E.M. (2001). Of mice and men: Comparison of the ultrastructure of megakaryocytes and platelets. Exp. Hematol..

[121] Balkenhol J., Kaltdorf K.V., Mammadova-Bach E., Braun A., Nieswandt B., Dittrich M., Dandekar T. (2020). Comparison of the central human and mouse platelet signaling cascade by systems biological analysis. BMC Genom..

[122] Ware J. (2004). Dysfunctional platelet membrane receptors: From humans to mice. Thromb. Haemost..

